# Behavioral correlates of health literacy among university students of health sciences in Kosovo: a cross-sectional study

**DOI:** 10.3325/cmj.2024.65.493

**Published:** 2024-12

**Authors:** Naim Jerliu, Haxhi Kamberi, Iris Mone, Pranvera Krasniqi, Genc Burazeri

**Affiliations:** 1 Faculty of Medicine, University of Prishtina, Prishtina, Kosovo; 2 National Institute of Public Health of Kosovo, Prishtina, Kosovo; 3 Faculty of Medicine, University of Gjakova “Fehmi Agani,” Gjakova, Kosovo; 4 Regional Hospital “Isa Grezda,” Gjakova, Kosovo; 5 Faculty of Medicine, University of Medicine, Tirana, Albania; 6 Department of International Health, CAPHRI (Care and Public Health Research Institute), Maastricht University, Maastricht, the Netherlands

## Abstract

**Aim:**

To assess the behavioral correlates of health literacy (HL) among university students of health sciences in Kosovo, irrespective of their sociodemographic characteristics.

**Methods:**

This cross-sectional study, carried out in Kosovo in 2024, enrolled 470 students of health sciences from the universities of Prishtina and Gjakova (86% women; mean age: 20.7 ± 2.7 years; response rate: 70%). We gathered HL data with the European Health Literacy Survey Questionnaire, as well as information on behavioral characteristics and sociodemographic factors. General linear model and binary logistic regression were used to assess the independent behavioral correlates of HL.

**Results:**

In multivariable-adjusted general linear models controlling for all sociodemographic factors and behavioral characteristics, lower HL scores were positively associated with alcohol consumption (36 vs 38 in non-drinkers, *P* = 0.024), low levels of physical exercise (36 vs 39 among students with high level of physical activity, *P* < 0.001), obesity (35 vs 39 among normal-weight individuals, *P* = 0.011), and a poorer health status (36 vs 37 among students with a better self-rated health status, *P* = 0.031). Furthermore, fully adjusted logistic regression models revealed a positive and strong relationship of “inadequate and/or problematic HL” (scores: 0-33) with physical inactivity (OR = 7.6, 95%CI = 1.8-31.9) and especially obesity (OR = 21.4, 95%CI = 3.8-119.8).

**Conclusions:**

Students with low HL may be more likely to engage in behaviors detrimental to health. There is need to enhance HL among future health professionals.

Health literacy (HL), generally referring to the ability to find, understand, and use health information for making appropriate health decisions (1,2), has been convincingly linked to health behaviors (3-6). Hence, lower HL levels have been linked to smoking (3,7,8) and smoking relapses (7,8), lower levels of physical activity (9-11) and its surrogates (3,10,11), alcohol use (11), unhealthy dietary habits (10,12), and a higher body mass (10).

Health outcomes of individuals are more influenced by their HL levels than by any other health determinants (13,14). From this perspective, low HL levels have been linked to poor quality of life and self-care (15), little health promotion skills (14), lack of use of health services when needed (16,17), and a poorer adherence to medical treatments (16,17).

Along with the interest in HL at a general population level, there is an increasing interest in HL among health professionals (14,18). In particular, there is a need to assess how health professionals understand HL from a professional standpoint and how this understanding influences their health practices (14). This is particularly important for students of health sciences, especially for nurses, who are expected to enhance patients’ HL skills (19).

More than 15 years after its independence, Kosovo is currently undergoing profound socio-political and economic transformations (20), which are also associated with changes in the burden of disease profile and behavioral patterns (20-22). As a matter of fact, Kosovo exhibits some of the poorest health indicators in Southeastern Europe (including life expectancy and child mortality), ranking lower than its neighboring countries (23). Similar to other low-and middle-income countries, the burden of non-communicable diseases, mainly of cardiovascular diseases, in Kosovo is increasing (23). Information on HL levels in the general population of Kosovo is scarce. The limited evidence is confined to a survey from 2011, which indicated low HL levels among individuals aged 65 years and above, especially those with a poorer self-perceived health status and chronic conditions (24). However, there is no information on the HL level and its distribution among current and future health professionals (students) in Kosovo.

In this context, we aimed to assess the behavioral correlates of HL among university students of health sciences in Kosovo, irrespective of a wide range of sociodemographic factors. In line with previous reports (3-6), we hypothesized a higher prevalence of unhealthy behaviors (including smoking, alcohol consumption, sedentary lifestyle, and obesity) among respondents with lower HL levels than in their counterparts with healthier behavioral practices.

## Respondents and methods

### Respondents and sampling

This cross-sectional study was conducted in Kosovo during February-April 2024. The study enrolled students attending one of the following study branches at the universities of Prishtina (capital of Kosovo) and Gjakova (one of the main regions of Kosovo): nursing, physiotherapy, public health, and health management. Of all eligible participants (N = 671), 113 (17% of the target population) did not complete the survey and further 88 (13% of the total) provided incomplete responses and were excluded from the analysis. There were no significant differences among respondents and non-respondents regarding sex and age distribution. The study sample included in the statistical analyses consisted of 470 students (405 women, or 86% of the overall sample). The final response rate was 470/671 = 70%.

### Instrument

All students who agreed to participate self-administered the structured questionnaire. Students were invited to fill in the questionnaires during classes by their respective lecturers. The questionnaire inquired about HL, selected behavioral and health characteristics, and sociodemographic factors.

HL was assessed with the internationally standardized European Health Literacy Survey Questionnaire (HLS-EU-Q) (25-27), which had been already validated in Albanian settings (28). The HLS-EU-Q consists of 47 items measuring students’ ability to access, understand, appraise, and apply health information across three health domains (health care, disease prevention, and health promotion) (26,27). Each item was rated on a 4-level Likert scale: “very easy” (score: 4), “fairly easy” (score: 3), “fairly difficult” (score: 2), and “very difficult” (score: 1). A summary score was calculated for each respondent and subsequently standardized on a scale ranging from 0 (lowest general HL level) to 50 (highest general HL level), according to the recommendations of developers of the instrument (25,29). Subsequently, the standardized summary scores of general HL were grouped into the following categories: “inadequate HL” (scores: 0-25.0), “problematic HL” (scores: >25.0-33.0), “sufficient HL” (scores: >33.0-42.0), and “excellent HL” scores (>42.0-50.0) (25,29).

Behavioral and health characteristics included smoking status (dichotomized into “yes” [including current and/or past smokers] vs “no” [never smokers]), alcohol consumption (dichotomized into “yes” vs “no” [never drinkers]), physical activity (trichotomized into “low,” “average,” and “high”), self-reported height and weight (based on which, body mass index [BMI] was calculated for each respondent and categorized into “normal” weight [BMI<25], “overweight” [BMI: 25-29.9], and “obesity” [BMI≥30]). There were no “thin” participants in the sample [BMI<19].), and self-rated general health status (dichotomized into: “good/excellent” vs “average/not good”).

Sociodemographic factors were sex (“men” vs “women”), age (18-19, 20, 21, and ≥22 years), institution (university of “Prishtina” vs “Gjakova”), branch of study (“nursing,” “health management,” “physiotherapy,” and “public health”), place of residence (“urban areas” vs “rural areas”), marital status (“single” vs “married”), current employment status (“yes” vs “no”), and economic level (“good” vs “not good”).

The study was approved by the Ethics Committee of the National Institute of Public Health of Kosovo and by the Ethics Committee of the Faculty of Medicine, University of Gjakova.

### Statistical analysis

A Fisher exact test was used to compare the distribution of behavioral and health characteristics between students with “sufficient/excellent” HL levels and those with “inadequate/problematic” HL levels (Table 1). A general linear model was used to compare the mean values of the summary scores of general HL (numerical term) between different categories of behavioral characteristics (Table 2). Crude (unadjusted) and multivariable-adjusted mean values and their respective 95% confidence intervals (CIs) and *P* values were calculated. Conversely, binary logistic regression was used to assess the association of general HL levels (dichotomized into “inadequate/problematic” HL vs sufficient/excellent” HL) with behavioral characteristics (Table 3). Crude (unadjusted) models were initially run (model 1). Subsequently, multivariable-adjusted models were run, while we controlled simultaneously for age and sex (model 2), all sociodemographic factors (model 3), and additionally for all behavioral factors (model 4). Crude/unadjusted and multivariable-adjusted odds ratios (ORs) and their respective 95% CIs and *P* values were calculated. A Hosmer-Lemeshow test was used to assess the goodness-of-fit of the multivariable-adjusted logistic regression models (30). A *P* value of ≤0.05 was considered statistically significant. SPSS, version 19.0 (IBM Corp., Armonk, NY, USA), was used for the statistical analysis.

**Table 1:** Distribution of health literacy (HL) by behavioral and health characteristics in a sample of university students of health sciences from Kosovo in 2024

Behavioral and health characteristics	Total (N = 470)	HL level (category)		
		Sufficient/excellent (N = 437)	Inadequate/problematic (N = 33)	P^†^
Smoking				
Yes	51 (10.9)*	41 (9.4)	10 (30.3)	0.001
No	419 (89.1)	396 (90.6)	23 (69.7)	
Alcohol consumption				
Yes	35 (7.4)	29 (6.6)	6 (18.2)	0.028
No	435 (92.6)	408 (93.4)	27 (81.8)	
Physical activity				
High	115 (24.5)	110 (25.2)	5 (15.2)	<0.001
Average	296 (63.0)	280 (64.1)	16 (48.5)	
Low	59 (12.6)	47 (10.8)	12 (36.4)	
BMI				
Normal	388 (82.6)	368 (84.2)	20 (60.6)	<0.001
Overweight	66 (14.0)	57 (13.0)	9 (27.3)	
Obese	16 (3.4)	12 (2.7)	4 (12.1)	
Self-rated health				
Good/Excellent	394 (83.8)	371 (84.9)	23 (69.7)	0.045
Average/Not good	76 (16.2)	66 (15.1)	10 (30.3)	

*Data are expressed as n (%).

**†**Fisher exact test.

**Table 2:** Association of health literacy (HL) score with students’ behavioral and health characteristics; mean values from the general linear models

Behavioral and health characteristics	Model 1*		Model 2^†^		Model 3^§^		Model 4^II^	
	Mean (95%CI)	P	Mean (95%CI)	P	Mean (95%CI)	P	Mean (95%CI)	P
Smoking								
Yes	39.5 (38.1-40.8)	0.062	39.1 (37.8-40.5)	0.058	37.2 (35.0-39.4)	0.018	36.3 (34.2-38.5)	0.264
No	40.8 (40.4-41.3)		40.5 (39.8-41.2)		39.0 (37.2-40.9)		37.2 (35.2-39.2)	
Alcohol consumption								
Yes	38.3 (36.7-39.9)	0.003	38.3 (36.7-39.9)	0.010	36.3 (33.9-38.7)	0.004	35.8 (33.4-38.1)	0.024
No	40.9 (40.4-41.3)		40.6 (39.9-41.3)		38.9 (37.0-40.7)		37.7 (35.8-39.6)	
Physical activity								
High	43.5 (42.7-44.4)	<0.001 (2) ^‡^	43.0 (42.1-43.9)	<0.001 (2)	41.3 (39.4-43.2)	<0.001 (2) ^‡^	39.1 (37.0-41.1)	**<0.001 (2)**
Average	39.8 (39.2-40.3)	<0.001 -	39.3 (38.6-40.0)	<0.001 -	37.8 (36.0-39.6)	<0.001 -	35.6 (33.6-37.6)	<0.001 -
Low	39.7 (38.5-40.9)	0.883	39.0 (37.7-40.3)	0.667	37.4 (35.4-39.4)	0.582	35.6 (33.4-37.8)	0.973
BMI								
Normal	41.2 (40.7-41.6)	<0.001 (2)	40.9 (40.2-41.6)	<0.001 (2)	39.8 (37.9-41.7)	<0.001 (2)	38.5 (36.5-40.4)	**<0.001 (2)**
Overweight	38.5 (37.3-39.7)	0.028 -	38.3 (37.1-39.5)	0.156 -	37.3 (35.3-39.2)	0.091 -	36.4 (34.4-38.4)	0.011
Obese	38.4 (36.0-40.8)	0.955	39.1 (36.7-41.6)	0.567	37.6 (34.7-40.5)	0.798	35.3 (32.5-38.2)	0.408
Self-rated health								
Good/Excellent	41.0 (40.5-41.5)	0.003	40.6 (39.9-41.2)	0.001	39.1 (37.2-40.9)	0.006	37.4 (35.4-39.3)	0.031
Average/Not good	39.2 (38.0-40.3)		38.6 (37.4-39.8)		37.4 (35.3-39.4)		36.1 (34.0-38.2)	

*Model 1: crude (unadjusted) models.

**†**Model 2: models adjusted for sex and age.

**‡**Overall *P* value and degrees of freedom (in parentheses).

§Model 3: models adjusted for all sociodemographic characteristics (sex, age, university, branch of study, residence, marital status, employment, and economic level).

IIModel 4: models adjusted additionally for all behavioral and health characteristics (smoking, alcohol consumption, physical activity, BMI, and self-rated health).

**Table 3:** Association of health literacy (HL) categories with behavioral and health characteristics of the students; results from binary logistic regression models

Behavioral and health characteristics	Model 1^†^		Model 2^‡^		Model 3^II^		Model 4^¶^	
	OR (95%CI) *	P	OR (95%CI)	P	OR (95%CI) ^a^	P	OR (95%CI)	P
Smoking:								
Yes	4.20 (1.87-9.43)	<0.001	3.34 (1.40-7.95)	0.006	4.73 (1.82-12.25)	0.001	2.49 (0.80-7.78)	0.117
No	1.00 (reference)		1.00 (reference)		1.00 (reference)		1.00 (reference)	
Alcohol consumption:								
Yes	3.13 (1.20-8.18)	0.020	2.04 (0.73-5.73)	0.174	2.44 (0.76-7.84)	0.135	1.92 (0.49-7.52)	0.351
No	1.00 (reference)		1.00 (reference)		1.00 (reference)		1.00 (reference)	
Physical activity:								
Low	5.62 (1.87-16.84)	<0.001 (2) ^§^	7.12 (2.25-22.54)	<0.001 (2)	6.94 (2.13-22.61)	<0.001 (2) ^§^	7.63 (1.83-31.85)	**0.001 (2)**
Average	1.26 (0.45-3.52)	0.002 -	1.42 (0.50-4.05)	<0.001 -	0.97 (0.33-2.90)	0.001 -	1.35 (0.38-4.82)	0.005 -
High	1.00 (reference)	0.663	1.00 (reference)	0.515	1.00 (reference)	0.958	1.00 (reference)	0.640
BMI:								
Obese	6.13 (1.82-20.73)	0.002 (2)	5.47 (1.43-20.95)	0.018 (2)	14.44 (3.08-67.77)	0.001 (2)	21.38 (3.82-119.8)	**0.002 (2)**
Overweight	2.91 (1.26-6.69)	0.004 -	2.24 (0.93-5.38)	0.013 -	2.36 (0.93-5.97)	<0.001 -	1.75 (0.63-4.86)	<0.001 -
Normal	1.00 (reference)	0.012	1.00 (reference)	0.071	1.00 (reference)	0.071	1.00 (reference)	0.280
Self-rated health:								
Average/Not good	2.44 (1.11-5.37)	0.026	2.87 (1.27-6.47)	0.011	2.67 (1.13-6.35)	0.026	2.31 (0.87-6.13)	0.094
Good/Excellent	1.00 (reference)		1.00 (reference)		1.00 (reference)		1.00 (reference)	

*Odds ratios (OR): “inadequate/problematic” HL vs “sufficient/excellent” HL.

†Model 1: crude (unadjusted) models.

‡Model 2: models adjusted for sex and age.

§Overall *P* value and degrees of freedom (in parentheses).

IIModel 3: models adjusted for all sociodemographic characteristics (gender, age, university, branch of study, residence, marital status, employment, and economic level).

¶Model 4: models adjusted additionally for all behavioral and health characteristics (smoking, alcohol consumption, physical activity, BMI, and self-rated health).

## Results

Overall, 25% of the students were 18-19 years old, and 19% were ≥22 years; 49% were from the University of Prishtina; 77% were nursing students; 42% were urban residents; and 33% reported a “not good” economic situation (data not shown).

The mean (SD) score of general HL was 41 ± 5 (on a scale from 0 to 50; not shown in the tables). Overall, 437 participants (almost 93% of the overall sample) exhibited “sufficient and/or excellent” HL levels, whereas the remaining 33 (7%) respondents displayed “inadequate and/or problematic” HL levels (Table 1). Compared with students with “sufficient and/or excellent” HL levels, students with “inadequate and/or problematic” HL levels had significantly higher prevalence of smoking (30% vs 9%; *P* = 0.001), alcohol consumption (18% vs 7%; *P* = 0.028), sedentary lifestyle (36% vs 11%; *P* < 0.001), obesity (12% vs 3%; *P* < 0.001), and a poorer self-rated health (30% vs 15%; *P* = 0.045).

The crude mean value of the general HL score (Table 2 – model 1) was lower among students who smoked (about 40 vs 41 in non-smokers, *P* = 0.062), those who drank alcohol (38 vs 41 in non-drinkers, *P* = 0.003), those who reported a sedentary lifestyle (40 vs 44 in those with high levels of physical activity, *P* < 0.001), obese students (38 vs 41 among normal-weight students, *P* = 0.028), and those with a poorer self-rated health (39 vs 41 in those with a better self-reported health status, *P* = 0.003). Adjustment for age and sex (model 2) did not alter the estimates. Adjustment for all sociodemographic characteristics (model 3) accentuated the relationship with smoking (37 vs 39 among non-smokers, *P* = 0.018), but attenuated the association with obesity (38 vs 40 among normal-weight students, *P* = 0.091). Upon additional adjustment for all behavioral characteristics (model 4), the association with smoking was not significant any longer, whereas the positive relationships of lower HL scores with alcohol consumption (36 vs 38 in non-drinkers, *P* = 0.024), low levels of physical exercise (36 vs 39 among students with high level of physical activity, *P* < 0.001), obesity (35 vs 39 among normal weight individuals, *P* = 0.011), and a poorer health status (36 vs 37 among participants with a better self-rated health status, *P* = 0.031) persisted (Table 2).

The crude (unadjusted) binary logistic regression models (Table 3 – model 1) showed strong positive associations between “inadequate and/or problematic” HL levels with smoking (OR = 4.2, 95%CI = 1.9-9.4), alcohol consumption (OR = 3.1, 95%CI = 1.2-8.2), low levels of physical activity (OR = 5.6, 95%CI = 1.9-16.8), obesity (OR = 6.1, 95%CI = 1.8-20.7), and a poorer self-rated health (OR = 2.4, 95%CI = 1.1-5.4). Upon adjustment for age and sex (model 2), the association with smoking was attenuated, the association with alcohol consumption was not significant any longer, whereas the association with physical activity was strengthened. Adjustment for all sociodemographic characteristics (model 3) accentuated the association of lower HL levels with smoking (OR = 4.7, 95%CI = 1.8-12.3) and particularly with obesity (OR = 14.4, 95%CI = 3.1-67.8). Upon further adjustment for all behavioral factors (model 4), there was no significant association of HL level with smoking (OR = 2.5, 95%CI = 0.8-7.8), alcohol consumption (OR = 1.9, 95%CI = 0.5-7.5), or self-rated health (OR = 2.3, 95%CI = 0.9-6.1), but there persisted a positive and strong relationship with low levels of physical activity (OR = 7.6, 95%CI = 1.8-31.9) and especially obesity (OR = 21.4, 95%CI = 3.8-119.8) (Table 3).

## Discussion

We found strong positive associations of lower HL levels with several unhealthy behavioral factors (including smoking, alcohol consumption, sedentary lifestyle, and obesity), irrespective of a range of sociodemographic characteristics of university students of health sciences in Kosovo. The associations were consistent (in both measuring scales of HL). Furthermore, the relationships with sedentary behavior and especially with obesity persisted strongly upon additional adjustment for all behavioral factors.

Our findings related to the positive association between lower HL levels and smoking are compatible with previous reports from the international literature (3,7,8). A recent study involving Spanish nursing students (3) reported that, in multivariable-adjusted logistic regression models, smokers were 38% less likely to have sufficient HL than non-smokers. Furthermore, a study conducted among African Americans indicated that low HL may be an independent risk factor for smoking (8). Thus, after adjusting for sociodemographic factors, participants with low HL were 68% more likely than those with high HL to be current smokers (8). In addition, previous studies linked low HL levels to smoking relapses (7,8). In our study, the association of low HL with smoking was strong and persisted upon adjustment for a range of sociodemographic characteristics but lost its statistical significance after adjustment for other behavioral factors.

Regarding alcohol consumption, we found a positive association with lower HL scores in general linear models controlling for all sociodemographic factors and behavioral characteristics, whereas in multivariable-adjusted logistic regression models the relationship of alcohol consumption with “inadequate/problematic” HL lost its statistical significance. Previous reports linked low HL levels with alcohol intake (11,31). However, a recent study in Spanish nursing students (3) did not find a significant independent association between alcohol use and inadequate HL. Nonetheless, alcohol use has been identified as part of a high-risk class (together with smoking, suicidal behavior, and unintentional injury), which has been linked to inadequate HL (11).

Our study showed a strong relationship between low HL and sedentary behavior, which persisted upon adjustment for all sociodemographic and health behavioral variables. This finding is in line with previous reports, which have convincingly linked low levels of physical activity to inadequate HL (9-11). In a comprehensive systematic review of observational studies, 18 of 22 studies found a significant positive association between high HL and physical activity (9). Conversely, in the only interventional study identified in this systematic review, HL was not a significant moderator of the intervention’s effectiveness (9). Also, in a recent study involving nursing students, sufficient HL was not related to greater physical activity, but was inversely related to sedentary lifestyle, evidenced by screen time (2). Furthermore, several other studies have linked surrogates of physical inactivity, including screen time to inadequate HL (10,11).

We found a particularly strong, consistent, and independent association of low HL with obesity. This is in line with previous studies (10,11), which linked inadequate HL with unhealthy dietary patterns and obesity. However, a recent study conducted among nursing students (3) did not find a significant association between diet quality and HL, speculating that nursing students generally have adequate knowledge about diet and, therefore, the perceived risk in the long term may not be a sufficient motivation for the adoption of healthy dietary patterns (3). In our study, we did not measure dietary patterns, but anthropometric indices only. The evidence that we obtained regarding the independent positive relationship between obesity and low HL is remarkable. Yet, there is a need for future studies among students of health sciences to replicate our findings.

As for the self-rated general health status, a previous study (8) has reported that, after controlling for a range of sociodemographic factors, low HL was significantly associated with poorer self-rated general health, which is compatible with our findings.

The prevalence of adequate (sufficient or excellent) HL in our study conducted in Kosovo is higher than the estimate reported in a cross-sectional study from the neighboring Albania. The Albanian study employed the same HL instrument in a sample of students attending different branches of health sciences (25). Also, our HL estimate is higher than that among nursing students in Namibia (14).

The overall prevalence of smoking found in our study (about 11%) was the same as that reported in a previous study conducted in Kosovo on a sample of individuals aged 18-23 years (32), but considerably lower than that reported in another study from Kosovo among individuals aged 18-24 (27%) (33). Regarding alcohol consumption, a previous study from Kosovo reported that 88% of young individuals never consumed alcohol or did so very rarely (32), which is comparable with our findings. There are no previous reports from Kosovo on the prevalence of obesity in the age group targeted in our study.

This study has several limitations, including potential selection bias from non-response and invalid information provided by nearly 30% of the targeted population. Some of the investigated categories may have been over- or underreported as respondents might have provided socially desirable answers, had recall inaccuracies, or misinterpreted questions. There are also limitations arising from the survey design, as associations observed in cross-sectional studies do not imply causality. Regardless of these potential drawbacks, our study provides useful evidence on important behavioral correlates of HL among future health professionals in Kosovo, a country striving to implement systemic changes and reforms needed for European Union membership.

In conclusion, our study indicates that students of health sciences with low HL levels may be more likely to engage in behaviors detrimental to health, which potentially undermines their future credibility as health professionals and role models. The main implications of this study include the necessity to integrate HL into curricula and implement support programs to improve students’ health behaviors. Addressing the HL gap can enhance the effectiveness of public health initiatives for combating the rising tide of obesity and sedentary habits, ultimately fostering more informed and healthier populations.
